# Expanding the Anti-Phl p 7 Antibody Toolkit: An Anti-Idiotype Nanobody Inhibitor

**DOI:** 10.3390/antib12040075

**Published:** 2023-11-16

**Authors:** Susan K. Vester, Anna M. Davies, Rebecca L. Beavil, Balraj S. Sandhar, Andrew J. Beavil, Hannah J. Gould, Brian J. Sutton, James M. McDonnell

**Affiliations:** Randall Centre for Cell and Molecular Biophysics, King’s College London, New Hunt’s House, London SE1 1UL, UK; susan.k.vester@kcl.ac.uk (S.K.V.); anna.davies@kcl.ac.uk (A.M.D.); rebecca.beavil@272bio.com (R.L.B.); b.s.sandhar@qmul.ac.uk (B.S.S.); andrew.beavil@kcl.ac.uk (A.J.B.); hannah.gould@kcl.ac.uk (H.J.G.); brian.sutton@kcl.ac.uk (B.J.S.)

**Keywords:** antibody, anti-idiotype, IgD, IgE, inhibitor, isotype, nanobody, Phl p 7

## Abstract

We have previously produced a toolkit of antibodies, comprising recombinant human antibodies of all but one of the human isotypes, directed against the polcalcin family antigen Phl p 7. In this work, we complete the toolkit of human antibody isotypes with the IgD version of the anti-Phl p 7 monoclonal antibody. We also raised a set of nanobodies against the IgD anti-Phl p 7 antibody and identify and characterize one paratope-specific nanobody. This nanobody also binds to the IgE isotype of this antibody, which shares the same idiotype, and orthosterically inhibits the interaction with Phl p 7. The 2.1 Å resolution X-ray crystal structure of the nanobody in complex with the IgD Fab is described.

## 1. Introduction

Antibodies are an important component of the human immune system, and failure or inappropriate response of the system can lead to disease, such as immunodeficiency [1], autoimmunity [2] or allergy [3]. In humans, there are five different antibody isotypes: IgA, IgD, IgE, IgG and IgM, of which two can be further divided into the subclasses IgA_1_, IgA_2_, IgG_1_, IgG_2_, IgG_3_ and IgG_4_. The different isotypes differ in their heavy chain sequence, length and structure, including length and flexibility of their hinge regions, the number of disulfide bonds and different glycosylation patterns [4,5]. Different antibody isotypes have different effector functions mediated by different receptors, as well as different tissue distributions and half-lives [5]. The site of antigen recognition in classical heterotetrameric antibodies is formed by the V_H_ and V_L_ domains; these domains produce the antibody idiotype, i.e., the sequence and structure of an antibody that confers its antigen specificity. Within the idiotype, the paratope is the physical binding site on the antibody that binds to the epitope on the antigen. It is mainly formed by the three complementarity-determining regions (CDRs) on each V_H_ and V_L_ domain, respectively, although with potential contributions from the framework region (FR) [6].

Human anti-Phl p 7 antibodies recognize the timothy grass (*Phleum pratense*) pollen allergen Phl p 7. Phl p 7 belongs to the polcalcin family and is a calcium-dependent 2-EF hand protein [7]. A human anti-Phl p 7 IgG_4_ antibody, 102.1F10, was identified in a patient undergoing grass pollen immunotherapy [8] and was used to develop a set of anti-Phl p 7 antibodies [9]. We have defined a nomenclature for the set of anti-Phl p 7 antibodies described in this study: the human anti-Phl p 7 IgG_4_ antibody is called HAPPIG_4_1. Other human anti-Phl p 7 antibody isotypes derived from clone 102.1F10 are termed HAPPIA1, HAPPID1, HAPPIE1 and HAPPIM1. These antibodies bind to Phl p 7 with subnanomolar affinities [8,10]. Besides binding to Phl p 7, cross-reactivity of the HAPPI1 antibodies to other polcalcin allergens from olive (Ole e 3), birch (Bet v 4) and alder tree (Aln g 4) has been observed, which provides a set of antigen (allergen) affinities from subnanomolar to low micromolar [8,11,12]. The HAPPI1 toolkit currently consists of eight antibody classes and subclasses, from four isotypes. IgA_1_/λ, IgA_2_/λ, IgE/λ, IgG_1_/λ, IgG_2_/λ, IgG_3_/λ, IgG_4_/λ and IgM/λ in the pVITRO1 vector were generated using the Polymerase Incomplete Primer Extension (PIPE) cloning method and are all available on Addgene [9]. The HAPPI1 antibody toolkit shares an identical V_H_ domain and light chain but differs in constant region heavy chain use. The toolkit can therefore be used as a model system for studying isotype differences and function. For example, HAPPIE1 has been used as a model system to study the effects of IgE affinity and valency in effector cell degranulation [12]. The crystal structure of the HAPPIG_1_1 Fab in complex with Phl p 7 has been solved [11].

Until now, the human antibody isotype IgD had been missing from the HAPPI1 toolkit. IgD is the least well-studied of the five human antibody isotypes, both in terms of its structure and function. For many decades, the functions of secreted IgD, first described in 1965 [13], remained largely unknown. However, recently emerging roles have been identified for secreted IgD in mucosal immunity [14] and allergic disease [15]. The crystal structure of the HAPPID1 Fab has recently been solved, allowing the first high-resolution analysis of the unique Cδ1 domain [16].

Nanobodies (Nbs) are derived from heavy-chain-only antibodies in camelids, which, unlike classical antibodies, contain their antigenic recognition potential within a single V_H_ domain (also termed V_H_H) [17]. Nbs are small (12–15 kDa), easy to make in bacterial expression systems and have emerged as valuable tools in research, diagnostics and therapy [18,19].

Here, we introduce the anti-IgD nanobody 072 (aδNb072), an orthosteric inhibitor of Phl p 7, for which we have determined the crystal structure in complex with the HAPPID1 Fab. The fifth isotype IgD completes the HAPPI1 antibody set and with aδNb072 adds an anti-idiotypic inhibitor to the toolkit.

## 2. Materials and Methods

### 2.1. Protein Expression and Purification

A pVITRO1-HAPPID1 construct (Addgene 204626) was cloned using PIPE [9,16], and a stable FreeStyle 293-F cell line was generated by hygromycin B selection, as previously described [10]. HAPPID1 was expressed in FreeStyle 293 Expression Medium (Thermo Fisher, Waltham, MA, USA) in spinner flasks, purified by affinity chromatography using its antigen Phl p 7 [16] and further purified by size exclusion chromatography on a Superdex 200 Increase 10/300 GL column (Cytiva, Marlborough, MA, USA) in PBS. A dot blot was performed for purified HAPPID1, with detection carried out using a goat anti-human IgD antibody HRP conjugate (Bethyl Laboratories, Montgomery, TX, USA) at 1:5000 dilution.

Two variants of the HAPPID1 Fab were produced. The first, lacking any purification tag, was expressed and purified as described previously [16] and was used for X-ray crystallographic studies. A second HAPPID1 Fab construct was produced with a C-terminal glycine_4_-serine linker and SpyTag [20] on the heavy chain and was used for interaction analyses. The SpyTagged versions of HAPPID1 Fab, HAPPIE1 Fab and HAPPID2 Fab were derived from pVITRO1-HAPPID1, pVITRO1-HAPPIE1 (Addgene 50365) [9] and pVITRO1-HAPPIE2 [12], respectively, using the NEBuilder HiFi DNA Assembly Cloning Kit (NEB, Ipswich, MA, USA). HAPPID1 Fab and HAPPIE1 Fab were expressed in Expi293F cells (Thermo Fisher) using the ExpiFectamine 293 Transfection Kit (Thermo Fisher) according to the manufacturer’s instructions. HAPPID2 Fab was expressed from stably transfected FreeStyle 293-F cells (Thermo Fisher) in DMEM supplemented with 10% (*v*/*v*) fetal bovine serum, 2 mM L-glutamine, 100 units/mL penicillin, 100 µg/mL streptomycin and 50 µg/mL hygromycin B. All SpyTagged Fabs were purified by SpySwitch affinity chromatography [21].

The pET151-Phl p 7 construct, with a C-terminal tryptophan residue to aid spectrophotometric detection [12], was expressed in BL21(DE3) in ZYP-5052 autoinduction medium [22] at 30 °C for 48 h. Phl p 7 was purified from whole cell lysate on a HisTrap FF Crude column and further purified by size exclusion chromatography on a Superdex 75 Increase 10/300 GL column (Cytiva), similarly to what has previously been described [11]. Phl p 7 was biotinylated using EZ-Link Maleimide-PEG_2_-Biotin (Thermo Fisher) according to the manufacturer’s instructions and excess biotin was removed by dialysis.

### 2.2. Development of Anti-IgD Nanobodies

Generation and identification of anti-IgD Nbs was performed by the VIB Nanobody Core (Vrije Universiteit Brussel, Ixelles, Belgium), as previously described [23,24]. In brief, a llama and an alpaca were each immunized with six injections of ~100 μg HAPPID1. Lymphocytes were prepared from anticoagulated blood collected from each animal four and eight days after the last HAPPID1 injection and total RNA was extracted. An independent Nb library from each animal was constructed using a 1:1 ratio of total RNA from the two timepoints. The libraries were separately panned on HAPPID1 and the output mixed for a further round of panning. Initial ELISA screening was used to validate binding of Nbs to HAPPID1.

### 2.3. Expression and Purification of aδNb072

For screening purposes, the pMECS-GG-aδNb072 construct, with a C-terminal HA tag and a His_6_-tag [25], was expressed in TG1 cells by IPTG induction as a protein III fusion protein. Periplasmic extracts were prepared using TES extraction [23].

The pET-15b-aδNb072 construct (Addgene 204627) was codon-optimized for *E. coli* expression and synthesized by GenScript. The construct contains a C-terminal TEV cleavage site and His_6_-tag. The pET-15b-aδNb072 construct was expressed in BL21(DE3) cells by IPTG induction at 18 °C overnight. After harvesting, bacterial pellets were frozen at −70 °C. Periplasmic extract containing aδNb072 was prepared by incubating thawed bacterial pellets in buffer A (10 mM phosphate, 500 mM NaCl, 2.7 mM KCl, 25 mM imidazole, 0.1% (*w*/*v*) NaN_3_, pH 7.4), supplemented with 250 units Benzonase (Merck, Boston, MA, USA) per L culture, for 45 min at room temperature on a roller. The periplasmic extract was clarified by centrifugation at 8900× *g* and 4 °C for 30 min. aδNb072 was purified on a HisTrap FF Crude column (Cytiva) with buffer A as wash buffer and eluted with buffer B (10 mM phosphate, 137 mM NaCl, 2.7 mM KCl, 500 mM imidazole, 0.1% (*w*/*v*) NaN_3_, pH 7.4) and then further purified by size exclusion chromatography on a Superdex 75 Increase 10/300 GL column (Cytiva).

### 2.4. Surface Plasmon Resonance

Surface plasmon resonance (SPR) molecular interaction analyses were performed using a Biacore T200 (Cytiva) in 10 mM HEPES pH 7.4, 150 mM NaCl, 0.05% (*v*/*v*) surfactant P20 as running buffer, supplemented with 5 mM CaCl_2_ for experiments involving Phl p 7. All experiments described here were performed at 25 °C.

An anti-His-tag antibody was immobilized onto a CM5 sensor chip using the His Capture Kit (Cytiva) according to the manufacturer’s instructions. Capture of 100 nM aδNb072 by an anti-His-tag antibody was performed at 10 μL/min for 180 s. For kinetic analyses, HAPPID1 and HAPPIE1 Fabs were injected at 20 μL/min for 240 s, with a dissociation phase of 900 s.

Biotinylated Phl p 7 was immobilized onto a streptavidin (SA) sensor chip (Cytiva). For capture, 50 nM HAPPID1 Fab was injected at 10 μL/min for 180 s. aδNb072 was flowed over at 20 μL/min for 240 s, with a dissociation phase of 300 s. For inhibition analysis, HAPPID1 Fab and aδNb072 were pre-complexed at 25 °C and injected at 20 μL/min for 240 s, with a dissociation phase of 300 s.

Regeneration was performed with 0.1 M glycine pH 2.0 for 60 s at 10 μL/min. Initial data analysis, including double-reference subtraction [26], was performed using Biacore T200 Evaluation software version 1.0 (Cytiva). Association and dissociation binding curves were plotted and fit using Origin 7 (OriginLab, Northampton, MA, USA). For fits of the association curves, data points were included until an association plateau was reached. k_off_ values were calculated using the in-built exponential decay fit in Origin 7. k_on_ values were derived by fitting k_obs_ using the equation y = B_eq_ × (1 − exp((−x) × (k_obs_)) and then plotting k_obs_ against concentration to estimate k_on_ from the slope of a linear fit [27]. K_D_ values were calculated using the ratio of k_off_/k_on_. Rate constants using a range of ligand concentrations from duplicate experiments were averaged and standard deviations calculated.

### 2.5. aδNb072/HAPPID1 Fab Complex Purification and Crystallization

To prepare the aδNb072/HAPPID1 Fab complex, the proteins were mixed with aδNb072 in excess and incubated overnight at 4 °C. The complex was then purified at room temperature by size exclusion chromatography using a Superdex 200 Increase 10/300 GL column that had been equilibrated with PBS containing 0.1% (*w*/*v*) sodium azide. Fractions containing the complex were pooled and concentrated. Crystals were grown at 18 °C in SWISSCI MRC 96-well plates using a reservoir volume of 100 μL and drops comprising 100 nL protein (A_280_ reading of 8.6) and 100 nL reservoir solution. The reservoir solution contained 15% (*w*/*v*) PEG 8000, 0.1 M sodium citrate and 0.05 M ammonium sulfate. The crystals grew with a needle-like morphology and were cryoprotected with 0.1 M sodium acetate pH 3.6, 28% (*w*/*v*) PEG 8000 and 10% (*v*/*v*) ethylene glycol before flash-cooling in liquid nitrogen.

### 2.6. X-ray Structure Determination and Refinement

X-ray diffraction data were collected at beamline I24 at the Diamond Light Source (Harwell, UK). Data were integrated using the DIALS data processing pipeline at Diamond [28] and further processed with programs from the CCP4 suite [29,30]. Datasets collected from two crystals grown in the same drop were merged. The data were anisotropic, with a resolution limit of 2.63 Å along the h axis. The structure was solved by molecular replacement with PHASER [31] using protein atoms from PDB entries 8OJT [16] and 4TVS [32] as search models. Structure refinement was performed with PHENIX [33] and manual model building with *Coot* [34]. Interfaces were analyzed with PISA [35]. Data processing and refinement statistics are presented in Table 1.

## 3. Results

### 3.1. Production of an Anti-Phl p 7 IgD Antibody

Until now, the HAPPI1 toolbox was incomplete, comprising eight antibody classes and subclasses, missing only the IgD isotype, HAPPID1. Consequently, we cloned HAPPID1 in pVITRO1 by swapping the antibody constant heavy chain using PIPE cloning [9,16]. The plasmid contains a dual antibody expression cassette, allowing HAPPID1 to be expressed from a single plasmid. The pVITRO1-HAPPID1 construct was transfected into FreeStyle 293-F cells, a stable cell line was generated, and HAPPID1 was expressed in FreeStyle 293 Expression Medium. We purified HAPPID1 by affinity chromatography using its antigen Phl p 7 and confirmed the identity of HAPPID1 by dot blot using an anti-human IgD HRP conjugate. The plasmid for this HAPPID1 antibody has been made available on Addgene.

After an additional size exclusion chromatography purification step, the HAPPID1 antibody was used for immunization of a llama and an alpaca for Nb generation, as described previously [24]. A library of anti-IgD Nbs was produced and characterized, and one specific anti-paratope Nb is described in this study.

### 3.2. Identification and Characterization of an Anti-Paratope Nb

**Figure 1:**
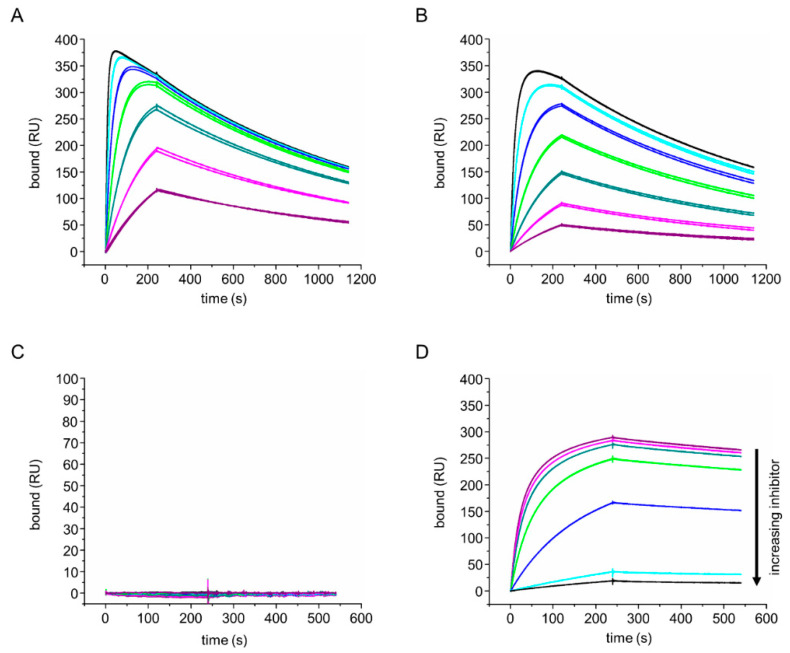
HAPPID1 Fab and HAPPIE1 Fab binding to aδNb072. (**A**,**B**) aδNb072 was captured on an anti-His-tag chip and (**A**) HAPPID1 Fab or (**B**) HAPPIE1 Fab was flowed over in a two-fold dilution series, with the highest concentration 200 nM Fab (black line) and the lowest concentration 3 nM Fab (purple line). (**C**) Biotinylated Phl p 7 was immobilized on an SA chip, HAPPID1 Fab was captured by Phl p 7 and aδNb072 was flowed over in a two-fold dilution series, with the highest concentration 200 nM aδNb072 (black line) and the lowest concentration 3 nM aδNb072 (purple line). (**D**) 50 nM HAPPID1 Fab was pre-complexed with decreasing concentrations of aδNb072 (50 nM in black, 40 nM in cyan, 30 nM in blue, 20 nM in green, 10 nM in dark cyan, 5 nM in magenta and 0 nM in purple) and flowed over immobilized Phl p 7. Data are shown in duplicate. RU, resonance units.

To identify Nb binding regions on IgD, we initially screened periplasmic extracts containing anti-IgD Nbs using SPR, before performing full concentration binding series using purified aδNb072. We captured anti-IgD Nbs via an anti-His-tag antibody and tested binding to HAPPID1, HAPPID1 Fab, HAPPIE1 Fab and HAPPID2 Fab. Within the Fab region, HAPPID1 and HAPPIE1 share the same V_H_ domain and light chain but differ in their C_H_1 domain (Cδ1 or Cε1). HAPPID2 Fab is another human-derived IgD Fab specific for Phl p 7 but has different V_H_ and light chain sequences [12]. We observed that aδNb072 bound to the HAPPID1 Fab with low nanomolar affinity (Table 2 and Figure 1A) and also bound the HAPPIE1 Fab (Table 2 and Figure 1B). aδNb072 did not bind to the HAPPID2 Fab (Supplementary Figure S1). The approximately 2.6-fold difference in affinity between HAPPID1 Fab and HAPPIE1 Fab was driven by a difference in the association rate constants, with an approximately 2.6-fold difference observed in k_on_, but nearly identical dissociation rate constants (Table 2).

Unusually, we observed a downturn in association curves at the highest concentrations for the HAPPID1 Fab or HAPPIE1 Fab interactions with captured aδNb072. This appears to be due to a small amount of induced dissociation [36], where Fab binding to aδNb072 slightly destabilizes the interaction between the aδNb072 His-tag and the capturing anti-His-tag antibody. Consequently, fits of association curves were only performed before binding plateaued (as shown in Supplementary Figure S2A). Plots of k_obs_ versus concentration showed the expected linearity for both HAPPID1 Fab and HAPPIE1 Fab (Supplementary Figure S2B,C), indicating that the induced dissociation phenomenon did not appear to impact k_obs_ substantially, even at high concentrations of analyte.

Using an SA chip with immobilized biotinylated Phl p 7, we found that HAPPID1 Fab captured by Phl p 7 was no longer able to bind to aδNb072 (Figure 1C). Using HAPPID1 Fab pre-complexed with aδNb072, we observed a concentration-dependent inhibition of binding to Phl p 7 (Figure 1D). These results indicate competition between the Phl p 7 and aδNb072 ligands, most likely due to overlapping binding sites. The structure of the aδNb072/HAPPID1 Fab complex, described below, demonstrates that aδNb072 is an orthosteric inhibitor of Phl p 7.

### 3.3. Interface between aδNb072 and the HAPPID1 Fab

**Figure 2:**
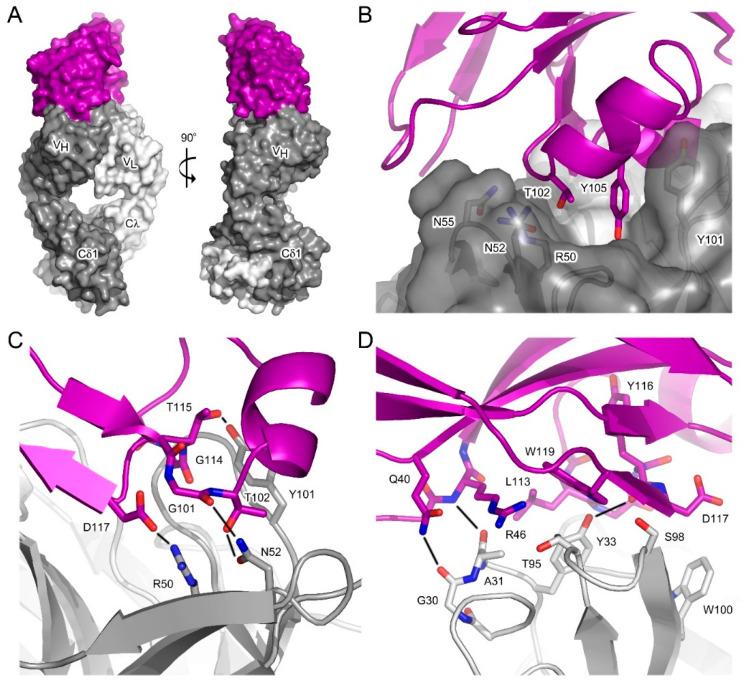
Crystal structure of the aδNb072/HAPPID1 Fab complex. (**A**) aδNb072 (purple) binds the HAPPID1 Fab V_H_ and V_L_ domains. Two views of the complex are shown; the second at a 90° anti-clockwise rotation relative to the first. (**B**) aδNb072 CDR3 residues T102 and Y105 bind in a depression created by CDRH1-3 from the Fab (dark gray). The depression is bordered at one end by the V_L_ domain (light gray). (**C**) The interface between aδNb072 and the V_H_ domain includes a number of hydrogen bonds and a salt bridge (all depicted by black lines). For clarity, the hydrogen bond between G114 (aδNb072) and S103 (Fab) is not shown. (**D**) The interface between aδNb072 and the V_L_ domain includes packing interactions between L113 (aδNb072) and Y33 (Fab), and W119 (aδNb072) and T95 (Fab), in addition to a number of hydrogen bonds (depicted by black lines). In panels (**A**–**D**), the V_H_ and V_L_ domains are colored in dark and light gray, respectively. aδNb072 is colored in purple.

The crystal structure of the aδNb072/HAPPID1 Fab complex was solved at 2.1 Å resolution, providing a high-resolution view of the interaction between these two binding partners (Figure 2A). The overall structure of the HAPPID1 Fab is similar to recently described structures [16] and will not be discussed in detail here. The interface between aδNb072 and the HAPPID1 Fab buries a surface area of ~840 Å^2^, of which most (~58%) of the binding surface is with the V_H_ domain. At the interface with the V_H_ domain (Figure 2B), CDR1 (aδNb072) contacts CDRH2 (Fab), CDR3 (aδNb072) packs against CDRH2 (Fab) and also forms extensive contacts with CDRH3 (Fab), while a minor contact is formed with CDRH1 (Fab). A notable feature of the interface is insertion of Thr102, Tyr105 and Asp117 (aδNb072, CDR3) in a depression created by CDRH1-3, which is bordered at one end by the V_L_ domain (Figure 2B). CDR3 from aδNb072 forms a number of hydrogen bonds with CDRH2 and CDRH3 from the Fab (Figure 2C): Gly101 (aδNb072)–Asn52 (Fab, CDRH2), Thr102 (aδNb072)–Asn52 (Fab, CDRH2), Gly114 (aδNb072)–Ser103 (Fab, CDRH3) and Thr115 (aδNb072)–Tyr101 (Fab, CDRH3). Moreover, the interface includes a salt bridge between Asp117 (aδNb072) and Arg50 (Fab, CDRH2).

At the interface with the V_L_ domain (Figure 2D), CDR3 and FR residues (aδNb072) contact CDRL1 (Fab); here, Leu113 (aδNb072, CDR3) packs against Tyr33 (Fab, CDRL1) and hydrogen bonds form between Gln40 (aδNb072, FR) and Gly30 (Fab, CDRL1), Arg46 (aδNb072, FR) and Ala31 (Fab, CDRL1), and Tyr116 (aδNb072, CDR3 mainchain) and Tyr33 (Fab, CDRL1). CDR3 (aδNb072) also contacts CDRL3 (Fab), and Trp119 (aδNb072), located at the junction between CDR3 and the FR, packs against Thr95 (Fab, CDRL3).

### 3.4. aδNb072 Is an Orthosteric Inhibitor of Phl p 7

**Figure 3:**
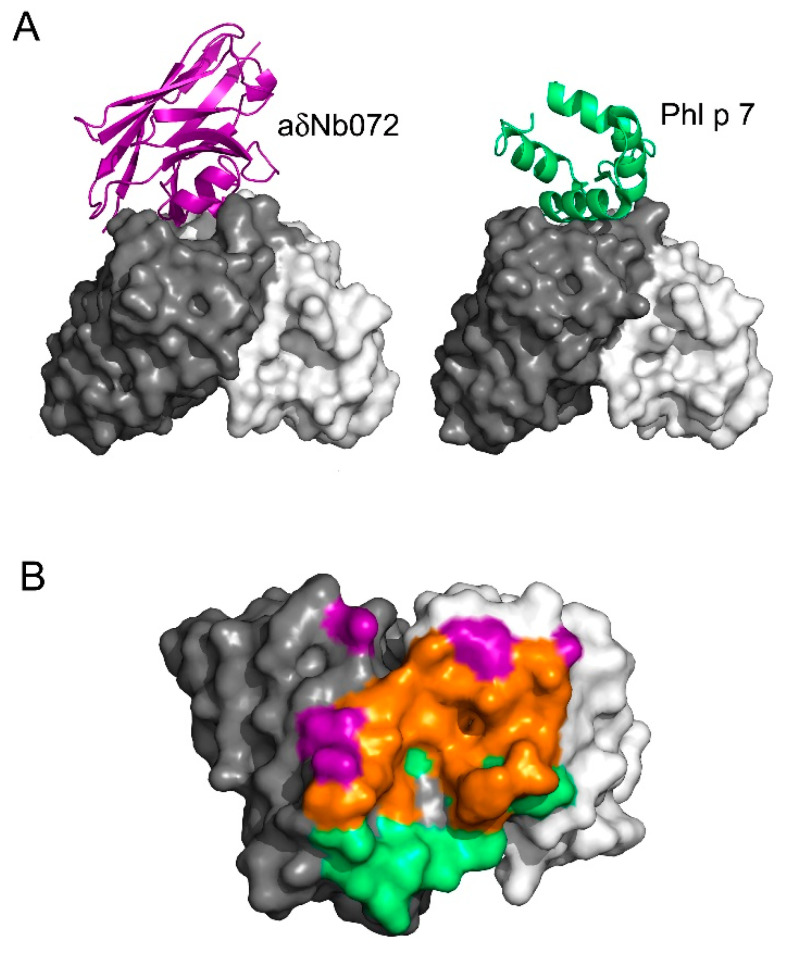
aδNb072 is an orthosteric inhibitor of Phl p 7. (**A**) The interactions between aδNb072 (purple) and Phl p 7 (green) and the V_H_ and V_L_ domains. (**B**) A view of the top of the V_H_ and V_L_ domains showing the aδNb072 and Phl p 7 paratopes. Residues that bind only aδNb072 are colored in purple, those that bind only Phl p 7 are colored in green and those that bind both aδNb072 and Phl p 7 are colored in orange. In panels (**A**,**B**), the V_H_ and V_L_ domains are colored in dark and light gray, respectively. For clarity, the C_H_1 and Cλ domains have not been shown.

Comparisons of the aδNb072/HAPPID1 Fab and Phl p 7/HAPPIG_1_1 Fab [11] complexes reveal substantial overlap between the aδNb072 and Phl p 7 binding sites (Figure 3). Approximately 70% of the Phl p 7 paratope on the V_H_ domain, and over 80% of the aδNb072 binding site, overlap. Both aδNb072 and Phl p 7 form substantial interactions with CDRH2 and CDRH3 on the Fab. Phl p 7 has a larger contact area with CDRH1 (~150 Å^2^), compared with aδNb072 (~22 Å^2^). Almost 100% of the Phl p 7 paratope on the V_L_ domain, and over 90% of the aδNb072 binding site, overlap, and both ligands contact CDRL1 and CDRL3 on the Fab.

## 4. Discussion

Here, we report the production of an anti-Phl p 7 IgD antibody, HAPPID1, and characterize aδNb072, a paratope-specific anti-idiotype Nb which binds to HAPPID1 and also HAPPIE1. The crystal structure of the aδNb072/HAPPID1 Fab complex reveals the Nb binding site and functional studies demonstrated competition between aδNb072 and the Phl p 7 antigen. Together, these data confirm an orthosteric mechanism of competition.

Antigen-binding regions of conventional antibodies are thought to form surfaces that range from concave to planar [37], while Nb paratopes are thought to be more convex, with a markedly protruding CDR3 loop, allowing easier recognition of cavities on the antigen surface [38,39]. While this is not always the case, the groove-like antigen-binding region of HAPPI1 may be an attractive target for Nbs. As seen here for aδNb072, the Nb CDR3 loop fits into the groove and space that is occupied by the Phl p 7 epitope when bound by HAPPID1. aδNb072 shows typical characteristics observed for Nbs, with antigen binding dominated by a long CDR3 loop, strong contributions from tyrosine residues as part of the Nb paratope, and the epitope recognized by the Nb (the HAPPID1 paratope) rich in aromatic residues [39]. Based on the structure of the aδNb072/HAPPID1 complex alone, it would not be possible to know whether the Nb was raised against the Fab or vice versa.

aδNb072 has a relatively fast association rate, which is likely to be electrostatically driven. A positively charged electrostatic patch located at the interface formed by V_H_ and V_L_ on HAPPID1 and a complementary negatively charged electrostatic patch on the Nb would be expected to drive the formation of the encounter complex [40]. It is noteworthy that aδNb072 had an increased association rate for the HAPPID1 Fab compared with the HAPPIE1 Fab, even though the amino acid sequences of the V_H_ and V_L_ domains are identical in the two isotypes. This suggests that the different C_H_1 domains in the two Fabs can affect the structure of the paratope, perhaps by producing small changes in the structure and dynamics of the interface between the V_H_ and V_L_ domains. Once bound, subtle conformational differences that affect the association of aδNb072 are no longer present and do not affect the dissociation rate. It is our expectation that aδNb072 interactions with other HAPPI1 isotypes might also show subtle differences compared to the HAPPID1 and HAPPIE1 interactions, but further experimental evidence is needed to confirm this. Two-fold differences in the binding affinity of Phl p 7 to different HAPPIG1 and HAPPIA1 subclasses have also previously been observed [10]. Indeed, there is a growing body of evidence pointing towards a role of the isotype or subclass in antigen recognition by imparting (subtle) structural changes that affect V_H_ and V_L_ domain conformation and/or relative orientation, and therefore antigen binding [6,41]. The mechanisms and potential implications of this phenomenon have not yet been fully elucidated, but the HAPPI1 system offers an excellent model for studying isotype differences and function.

Anti-idiotype Nbs or anti-idiotype antibodies can be paratope-specific, entirely or partially, or non-paratope specific. aδNb072, described here, is paratope-specific, able to block the binding of Phl p 7 to HAPPID1 and HAPPIE1. Anti-idiotype antibodies have a long history, and the idea that they may act as molecular mimics of antigenic epitopes has been explored for vaccine or immunotherapy applications, albeit with limited success [42]. In this case, the ligands aδNb072 and Phl p 7 share no significant structural similarity, either in terms of backbone element or conserved contact residues.

aδNb072 as an inhibitor preventing Phl p 7 binding to HAPPIE1 (or other HAPPI1 classes) may be a useful tool for functional studies and beyond. In allergic disease, cross-linking of IgE bound to its high-affinity receptor FcεRI on the surface of mast cells or basophils by antigen (allergen) initiates an immune response, i.e., effector cell degranulation [43]. In vitro, HAPPIG1 and HAPPIA1 subclasses have been able to inhibit Phl p 7-mediated HAPPIE1-dependent basophil activation [8,10]. While we would not expect aδNb072 to bind to other anti-Phl p 7 idiotypes that might additionally be found in vivo, and no binding was observed to HAPPID2, this Nb is a valuable tool as part of the HAPPI1 model system.

In conclusion, we have characterized aδNb072 as an anti-paratope inhibitor, which blocks binding of Phl p 7 to HAPPI1, and determined the crystal structure of aδNb072 in complex with HAPPID1 Fab, adding both aδNb072 and HAPPID1 to the anti-Phl p 7 antibody toolkit.

## Figures and Tables

**Table 1:** X-ray data processing and refinement statistics.

	aδNb072/HAPPID1 Fab
**Data Processing**	
Space group	*P* 2_1_ 2_1_ 2_1_
*a*, *b*, *c* (Å)	65.74, 71.04, 122.17
Resolution (Å) ^a^	61.09–2.10 (2.16–2.10)
Completeness (%) ^a^	100.0 (100.0)
Multiplicity^a^	26.4 (26.7)
Mean (I)/σ (I)^a^	9.5 (1.2)
CC_1/2_^a^	0.998 (0.611)
*R*_pim_ (%) ^a^	6.2 (65.5)
Wilson *B* factor (Å^2^)	35.23
**Refinement**	
R_work_/R_free_ (%) ^b^	21.42/25.92
No. of reflections	34 098
RMSD	
Bond lengths (Å)	0.002
Bond angles (°)	0.555
Coordinate error (Å)	0.26
No. of atoms	
Protein	4 281 ^c^
Solvent	148
Other	49 ^d^
Average B factor (Å^2^)	
Protein	49.15
Solvent	42.97
Other	52.98
Ramachandran plot	
Favored (%)	97.51
Allowed (%)	2.49

^a^ Values in parentheses are for the outer shell; ^b^ R_free_ set comprises 5% of reflections; ^c^ Includes alternative conformations; ^d^ Acetate, ethylene glycol and sodium.

**Table 2:** Binding characteristics of aδNb072 to HAPPID1 Fab and HAPPIE1 Fab.

	HAPPID1 Fab	HAPPIE1 Fab
K_D_ ± SD (M)	1.40 (±0.01) × 10^−9^	3.64 (±0.08) × 10^−9^
k_on_ ± SD (M^−1^ s^−1^)	5.86 (±0.01) × 10^5^	2.28 (±0.01) × 10^5^
k_off_ ± SD (s^−1^)	8.22 (±0.08) × 10^−4^	8.29 (±0.20) × 10^−4^

Experiments were performed in duplicate; data are given as ± standard deviation (SD).

## Data Availability

The pVITRO1-HAPPID1 and pET-15b-aδNb072 constructs have been deposited in the Addgene repository. Coordinates and structure factors for the aδNb072/HAPPID1 Fab complex have been deposited in the Protein Data Bank with accession number 8Q6K.

## Supplementary Materials

The following supporting information can be downloaded at: https://www.mdpi.com/article/10.3390/antib12040075/s1, Figure S1: HAPPID2 Fab binding to aδNb072; Figure S2: SPR data analysis and fitting.
