# Supplementary material for: Expanding the Anti-Phl p 7 Antibody Toolkit: An Anti-Idiotype Nanobody Inhibitor
Source: Antibodies (Basel). 2023 Nov 16;12(4):75. doi: 10.3390/antib12040075 (PMC10660547; doi:10.3390/antib12040075)
Supplement: Supplementary file 1 [file antibodies-12-00075-s001.zip › antibodies-2590729-supplementary.pdf]

Supplementary Materials

Expanding the Anti-Phl p 7 Antibody Toolkit: An Anti-Idiotype Nanobody Inhibitor

Susan K. Vester [1], Anna M. Davies [1], Rebecca L. Beavil [1,2], Balraj S. Sandhar [1,3], Andrew J. Beavil [1], Hannah J. Gould [1], Brian J. Sutton [1] and James M. McDonnell [1,*]

[1] Randall Centre for Cell and Molecular Biophysics, King's College London, New Hunt's House, London SE1 1UL, UK
[2] Current address: 272BIO Limited, The Pirbright Institute, B-Block, Ash Road, Pirbright, Woking, Surrey GU24 0NF, UK
[3] Current address: William Harvey Research Institute, Barts and The London School of Medicine and Dentistry, Queen Mary University of London, London EC1M 6BQ, UK

*Corresponding author (james.mcdonnell@kcl.ac.uk)

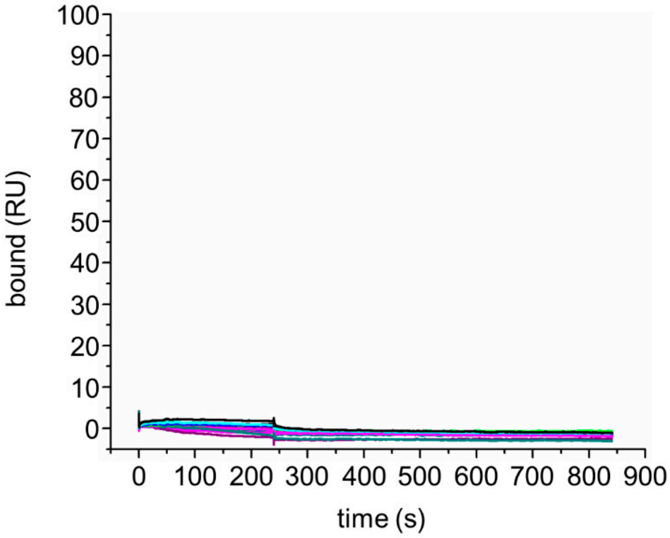

Supplementary Figure S1: HAPPID2 Fab binding to aδNb072. aδNb072 was captured on an anti-His-tag chip and HAPPID2 Fab was flowed over in a two-fold dilution series, with the highest concentration 200 nM HAPPID2 Fab (black line) and the lowest concentration 3 nM HAPPID2 Fab (purple line), as in Figure 1. Data are shown in duplicate. RU, resonance units.

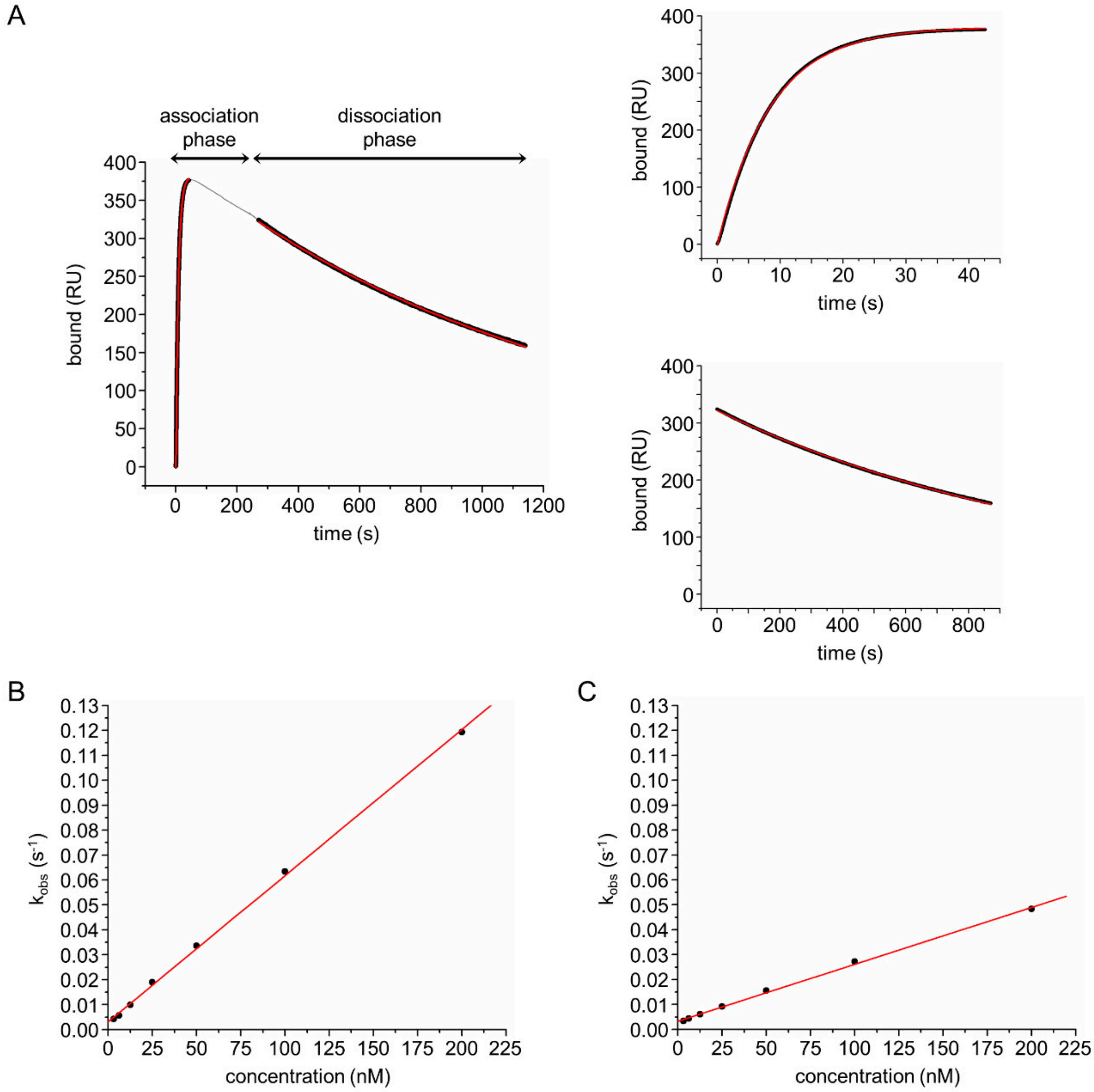

Supplementary Figure S2: SPR data analysis and fitting. (A) Example data from Figure 1A showing 200 nM HAPPID1 Fab flowed over aδNb072 captured via an anti-His-tag antibody (gray line). Data ranges that were used for data fitting are shown in black, with the fit of the data shown in red. The right-hand panels illustrate the extracted data used for fitting k_{obs} (top panel) and k_{off} (bottom panel). (B) k_{obs} values (black circles), derived from fitting the association curves of HAPPID1 Fab binding to aδNb072, were plotted against concentration of HAPPID1 Fab to estimate k_{on} from the slope of the linear fit (red line). (C) k_{obs} values (black circles), derived from fitting the association curves of HAPPIE1 Fab binding to aδNb072, were plotted against concentration of HAPPIE1 Fab to estimate k_{on} from the slope of the linear fit (red line).
